# Efficacy of modified thoraco-laparoscopic Ivor-Lewis versus traditional thoraco-laparoscopic Ivor-Lewis for esophageal cancer: Propensity score-matched analysis

**DOI:** 10.3389/fonc.2022.1076014

**Published:** 2023-01-17

**Authors:** Ziqiang Hong, Wenxi Gou, Yingjie Lu, Xusheng Wu, Yannan Sheng, Baiqiang Cui, Xiangdou Bai, Dacheng Jin, Yunjiu Gou

**Affiliations:** ^1^ The First Clinical Medical College of Gansu University of Chinese Medicine, Gansu Provincial Hospital, Lanzhou, China; ^2^ Department of Thoracic Surgery, Gansu Provincial Hospital, Lanzhou, China; ^3^ School of Public Health, Southern Medical University, Guangzhou, China

**Keywords:** esophageal cancer, Ivor-Lewis, thoraco-laparoscopy, propensity score matching analysis, modified

## Abstract

**Objective:**

To compare the clinical results of the modified Ivor-Lewis procedure, which preserves the azygous vein, thoracic duct and surrounding tissues, with the traditional Ivor-Lewis procedure, which removes these tissues, for treating esophageal squamous cell carcinoma, and evaluating whether the azygous vein, thoracic duct and surrounding tissues are required to be removed for the surgery of esophageal cancer.

**Methods:**

To retrospectively analyze the clinical data of patients suffering from esophageal cancer treated by thoracic-laparoscopic Ivor-Lewis procedure admitted to the Department of Thoracic Surgery of Gansu Provincial People’s Hospital from September 2017 to September 2019. According to the surgical method, they were divided into the modified thoracolaparoscopic Ivor-Lewis (modified group) and the traditional thoracolaparoscopic Ivor-Lewis (traditional group). Propensity score matching analysis (PSM) was applied to reduce the selection bias of confounding factors.

**Results:**

A total of 245 patients who suffered from esophageal cancer and underwent thoracic-laparoscopic Ivor-Lewis were enrolled in the study. There were 124 cases in the modified group and 121 cases in the traditional group. The discrepancies in the age and T-stage among patients in the traditional and modified groups were statistically significant. After PSM, the above-mentioned factors became statistically insignificant. There were 86 patients in each group after PSM. Compared with the traditional group, the modified group has shorter operative time (*p*=0.007), less intraoperative bleeding (*p*=0.003) and less postoperative 3 days chest drainage(*p*=0.001), with a statistically significant difference. No significant difference in local recurrence (*p*=0.721) and distant metastasis (*p*=0.742) after surgery were found in the two groups, and the difference was not statistically significant. There was also no statistically significant difference in the 3-year postoperative survival rate (44.2% vs. 41.9%, *p*=0.605) between the modified and traditional groups.

**Conclusion:**

The modified Ivor-Lewis procedure, which preserves the azygous vein, thoracic duct, and surrounding tissue, reduces surgical trauma in esophageal cancer, has not increased postoperative recurrent metastases, while achieved the same long-term outcomes as expanded surgery.

## Introduction

Esophageal cancer is one of the most aggressive gastrointestinal malignancies (1), with an overall 5-year survival rate between 15% and 25% worldwide, and is the sixth leading cause of cancer-related deaths worldwide (2). In the era of multimodality treatment, esophagectomy remains the primary treatment for the patients with resectable esophageal cancer. Since the first report by Lewis-sanby (3) in 1946, the Ivor-Lewis procedure of esophageal cancer has progressively become the standard surgical approach for treating esophageal cancer in Europe and the United States owing to the fact that it agrees with the principles of surgical treatment of malignant tumors by removing the esophageal tumor and surrounding tissues in a whole block. However, the wide resection area of the Ivor-Lewis procedure requires whole resection of the esophageal tumor and its surrounding mediastinal tissues, including the esophagus and surrounding lymphatic fatty tissues, bilateral mediastinal pleura, and part of the pericardium; and also requires resection of the posterior esophagus, azygous vein, thoracic duct, and surrounding tissues, it is highly traumatic and has more postoperative complications, and is slowly carried out in China (4, 5). In recent years, with the extensive application of thoracoscopic techniques, the combined thoracolaparoscopic esophagectomy has become the mainstream surgical treatment of esophageal cancer in China (6, 7). In thoracoscopic esophageal cancer resection, the azygous vein, thoracic duct and surrounding tissues are preserved, and it remains unclear whether the reduction of surgical extent will increase the risk of postoperative recurrence and decrease the long-term outcome. Therefore, the purpose of present study was to compare the clinical results of the modified Ivor-Lewis procedure, which preserves the azygous vein, thoracic duct and surrounding tissues, with the classical Ivor-Lewis procedure, which removes these tissues, for the treatment of esophageal squamous cell carcinoma, in order to provide more options for the improvement of the esophageal cancer procedure.

## Materials and methods

This study has been reviewed by the Ethics Committee of Gansu Provincial People’s Hospital, approval number: 2022-350. All patients signed the informed consent form for surgery before surgery.

### Clinical information

Clinical data of patients with esophageal squamous carcinoma admitted to the Department of Thoracic Surgery of Gansu Provincial People’s Hospital undergoing surgical treatment from September 2017 to September 2019 were collected. The group was divided into traditional group and modified group according to the different intraoperative surgical techniques, with 121 patients undergoing traditional thoracolaparoscopic Ivor-Lewis surgery from September 2017 to September 2018 and 124 patients undergoing modified thoracolaparoscopic Ivor-Lewis surgery from October 2018 to September 2019.

Inclusion criteria: (i) clear diagnosis of esophageal squamous carcinoma by preoperative thoracic and abdominal computed tomography (CT), gastroscopy, pathological biopsy and upper gastrointestinal imaging; (ii) American Joint Committee on Cancer(AJCC) 8th edition pathological staging of T1-T3, as well as partially resectable T4a; no distant metastasis (M0) detected by preoperative examination and intraoperative exploration, clinical stage I-III; (iii) no previous history of esophageal and gastric surgery; (iv) postoperative pathology suggesting negative stump and confirmed R0 resection; (v) complete clinical data (complete surgical records, pathological data and postoperative follow-up data).

Exclusion criteria: (i) patients unable to tolerate surgery due to advanced age, cardiopulmonary or hepatic or renal insufficiency; (ii) previous history of tumor or combination of malignant tumors from other sites; (iii) lost to follow-up time or death due to other reasons.

All enrolled patients were divided into two groups: the modified group with preserving the azygous vein, thoracic duct and surrounding tissues during esophagectomy, and the traditional group with resecting above tissues. All thoraco-laparoscopic Ivor-Lewis procedures were performed by the same thoracic surgeon (Prof. Yunjiu Gou).

### Surgery method

**Figure 1:**
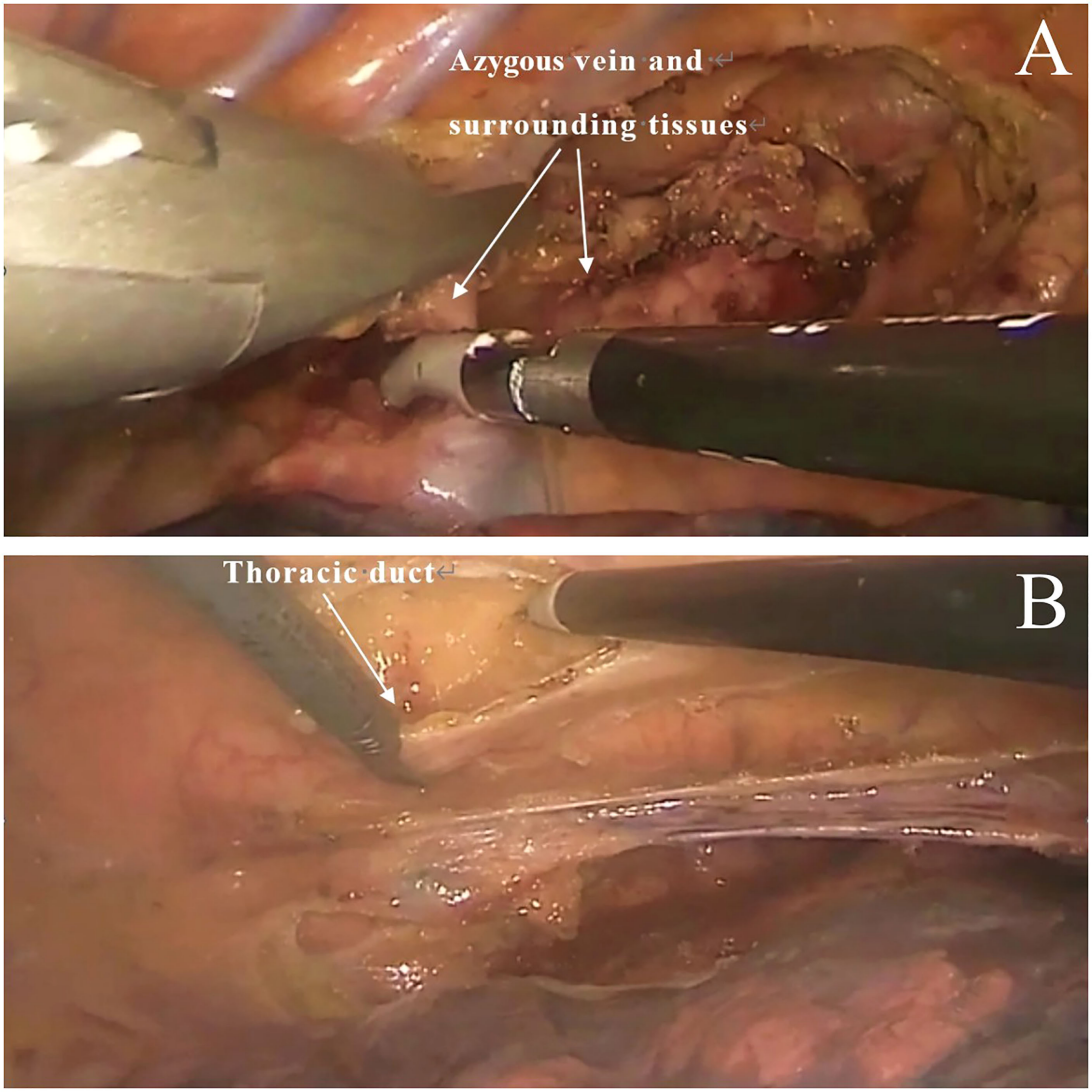
Traditional technique. **(A)** Excision of the azygous vein and surrounding tissues. **(B)** Excision of thoracic duct.

**Figure 2:**
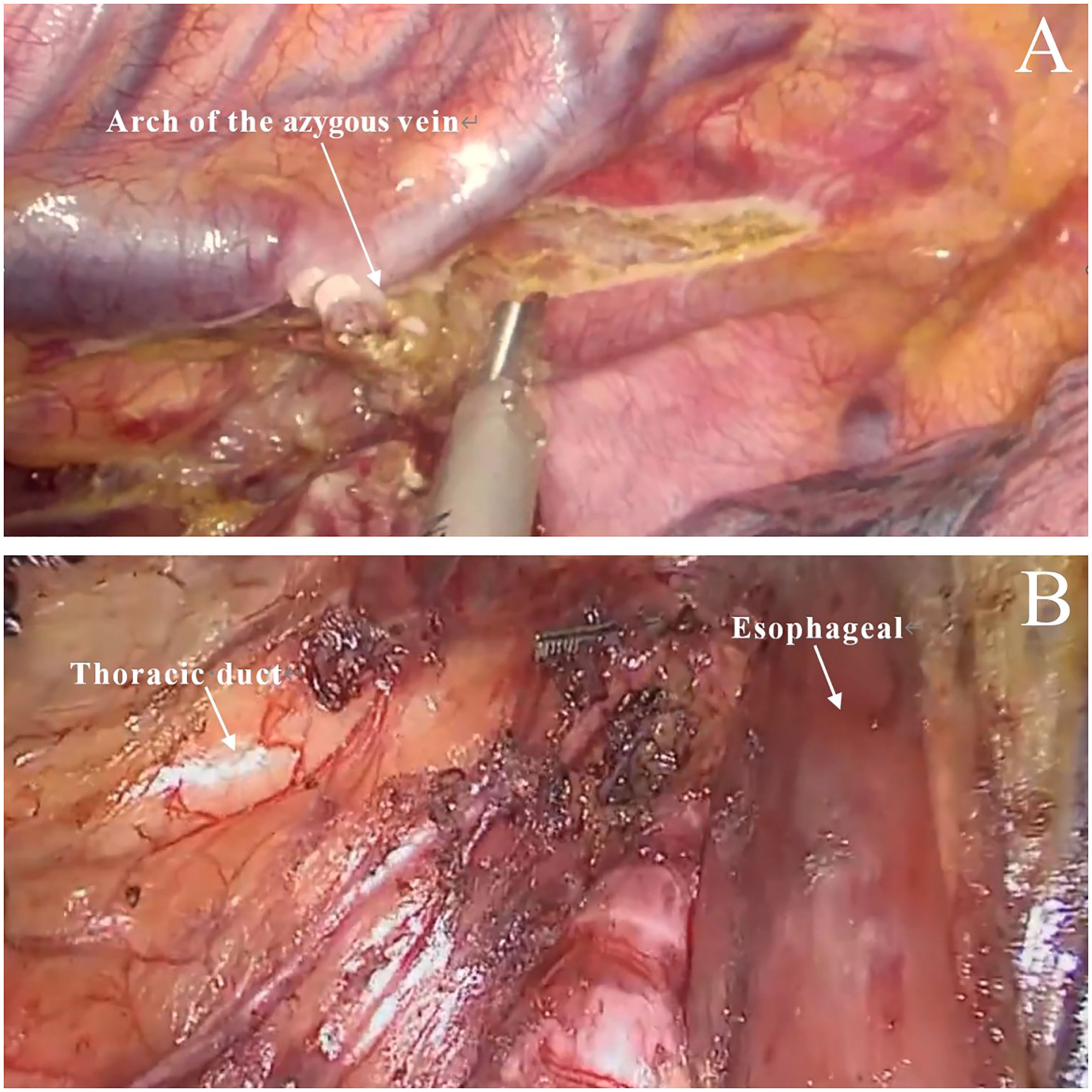
Modified technique. **(A)** Only the arch of the azygous vein was cut (the posterior esophagus, azygous vein and surrounding tissues were preserved). **(B)** The thoracic duct and surrounding tissues were preserved.

Patients with squamous carcinoma of the upper thoracic segment of the esophagus and CT suggestive of lymph node enlargement in the neck were treated with a three-incision esophagectomy from the mid-upper abdomen-right posterior lateral thorax-neck; the rest of patients with lesions in the middle and lower thoracic segments were treated with a two-incision esophagectomy from the mid-upper abdomen-right posterior lateral thorax. Chest surgery operations: (i) the traditional group: the whole esophagus and its adjacent tissues including the thoracic duct and the azygous vein between the spine and the pericardium were removed by a posterior lateral incision into the right thorax at the 5th rib, and the lymph nodes in the azygous vein, thoracic duct and surrounding tissues were dissected separately after surgery and sent for examination (**Figure 1**). The lymph nodes beside the esophagus, main pulmonary artery, carina, and recurrent laryngeal nerve were cleared, and then the stomach and esophagus were anastomosed at the top of the chest. (ii) the modified group: only the arch of the azygous vein was cut, the posterior esophagus, azygous vein, thoracic duct and surrounding tissues were preserved (**Figure 2**), and the rest of the chest operation was same as the traditional group. The abdominal and neck surgical operations were same for both groups of patients. Abdominal surgical operations: the stomach was freed after exploration through a median epigastric incision, and the epigastric region of lymph nodes was cleared and a tubular stomach was formed. Surgical operations on the neck (triple incision patients): left/right cervical sternocleidomastoid muscle anterior margin incision, free esophagus, selective lymph node removal, transesophageal bed lift of the tubular stomach, and esophagogastric anastomosis.

### Observation indicators

(i) Baseline information: sex, age, body mass index (BMI), tumor size, history of alcohol consumption, tumor location, pT stage, pN stage and pathological stage; (ii) surgical data: including total operating time (from the beginning of skin incision to the completing of sutured incision), intraoperative bleeding, number of lymph nodes dissected, postoperative chest drainage for 3 days, postoperative hospital stay and postoperative complications; (iii) prognosis: follow-up from the day of operation to the postoperation of 36 months.

### Diagnosis and follow-up of postoperative recurrence

The diagnosis of postoperative recurrence is based on patient history, physical examination, imaging and pathological examination. All patients were reviewed every 3 months for the first 3 years after surgery, every 6 months after 3 years until 5 years, and annually thereafter (8). Follow-up review of enhanced CT of the chest and abdomen, and gastroscopy 1 year after surgery. Patients presenting clinical signs and symptoms should be followed up promptly. The site and time of recurrence was based on the date of imaging or (and) pathological diagnosis.

### Statistical analysis

Data were analyzed using SPSS 26.0 software (SPSS Inc., Chicago, IL, USA), with continuous variables expressed as mean ± standard deviation ( $\bar{\text{x}}$ ± s) and t-test was employed to two independent samples for comparison; categorical variables were described as frequencies and percentages (%), and the chi-square test or Fisher test was applied to compare the results among groups. PSM analysis was performed using R 4.1.1, and patients were matched on the basis of PSM by using the nearest neighbor method with a matching ratio of 1:1 and a caliper value was set to be 0.02 (without replacement). Standardized mean differences (SMD) was used to evaluate the covariate balance before and after matching. A good covariate balance was presented as the SMD < 0.1. Survival curves were plotted using R 4.1.1 and the survminer package with the Kaplan-Meier method and log-rank tests. Herein, *p*<0.05 was considered to be a statistically significant difference.

## Results

### Demographic and clinical characteristics

Before PSM, the difference of age and T stage between the two groups were statistically significant. There were 86 patients in each group after PSM. The distribution of baseline characteristics were balanced between the two groups. No differences were observed between the modified group and the traditional group in terms of sex, age, BMI, tumor size, drinking history, tumor location, pT stage, pN stage and pathological stage (**Table 1**).

**Table 1:** Comparison of baseline information before and after propensity score matching between the two groups [cases (%)/ $\bar{x}$ ± *s*].

Characteristic	Before PSM			After PSM		
	Modified group (n=124)	Traditional group (n=121)	*P* (SMD)	Modified group (n=86)	Traditional group (n=86)	*P* (SMD)
Sex			0.376 (0.036)			0.151 (0.058)
Male	87 (70.2)	91 (75.2)		51 (59.3)	60 (69.8)	
Female	37 (29.8)	30 (24.8)		35 (40.7)	26 (30.2)	
Age (years)	61.52 ± 4.07	62.69 ± 4.45	0.032 (0.453)	60.40 ± 4.15	61.06 ± 4.79	0.299 (0.037)
BMI(kg/m^2^)	22.95 ± 1.76	22.65 ± 2.07	0.224 (0.030)	23.23 ± 2.34	22.90 ± 2.27	0.312 (0.021)
Tumor size (cm)	3.19 ± 0.38	3.20 ± 0.37	0.713 (0.042)			
Drinking History			0.555 (0.018)			0.357 (0.006)
Yes	64 (51.6)	67 (55.4)		41 (47.7)	35 (40.7)	
No	60 (48.4)	54 (44.6)		45 (52.3)	51 (59.3)	
Tumor location			0.369 (0.008)			0.401 (0.012)
Upper thoracic	17 (13.7)	11 (9.1)		17 (19.8)	11 (12.8)	
Middle thoracic	84 (67.8)	81 (66.9)		48 (55.8)	49 (57.0)	
Lower thoracic	23 (18.5)	29 (24.0)		21 (24.4)	26 (30.2)	
pT Staging			0.027 (0.362)			0.086 (0.063)
T_1_	17 (13.7)	13 (10.7)		17 (19.8)	7 (8.1)	
T_2_	25 (20.2)	43 (35.6)		23 (26.7)	28 (32.6)	
T_3_	82 (66.1)	65 (53.7)		46 (53.5)	51 (59.3)	
pN Staging			0.388 (0.023)			0.106 (0.031)
N_0_	56 (45.2)	62 (51.2)		41 (47.7)	37 (43.0)	
N_1_	42 (33.8)	29 (24.0)		33 (38.4)	26 (30.2)	
N_2_	24 (19.4)	27 (22.3)		12 (13.9)	23 (26.8)	
N_3_	2 (1.6)	3 (2.5)		0	0	
Pathological staging			0.432 (0.065)			0.312 (0.052)
I	17 (13.7)	17 (14.1)		17 (19.8)	10 (11.6)	
II	43 (34.7)	51 (42.1)		24 (28.0)	29 (33.7)	
III	64 (51.6)	53 (43.8)		45 (52.2)	47 (54.7)	

BMI, body mass index; red text indicates SMD, standardized mean differences.

### Comparison of surgical data and postoperative recovery between the two groups

The surgical data of the two groups of patients after PSM are specified in **Table 2**. By comparing the surgical data and postoperative complications of the two groups, it was found that the total operative time(*p*=0.007), intraoperative bleeding(*p*=0.003), and postoperative 3 days chest drainage(*p*=0.001) were less in the modified group than that in the traditional group, with statistically significant differences. However, the differences in postoperative complications between the two groups were not statistically significant.

**Table 2:** Surgical data of patients [cases (%)/ $\bar{x}$ ± *s*].

Characteristic	Modified group (n=86)	Traditional group (n=86)	*p*
Total operation time (min)	201.63 ± 25.11	212.21 ± 26.00	0.007
Intraoperative bleeding volume (ml)	141.05 ± 24.69	152.91 ± 27.35	0.003
Number of lymph node dissection	29.54 ± 2.14	29.64 ± 2.23	0.754
Postoperative 3 days chest drainage (ml)	888.95 ± 148.80	975.00 ± 179.34	0.001
Postoperative hospital stay (d)	10.60 ± 1.64	11.87 ± 2.31	<0.001
Postoperative complications			
Pulmonary infection	4 (4.7)	5 (5.8)	0.732
Anastomotic fistula	1 (1.2)	2 (2.3)	0.560
Anastomotic stenosis	4 (4.7)	3 (3.5)	0.700
Chylothorax	0	0	–
Difficulty swallowing	6 (7.0)	5 (5.8)	0.755
Recurrent laryngeal nerve injury	2 (2.3)	3 (3.5)	0.650

### Recurrence of metastasis and long-term survival

**Figure 3:**
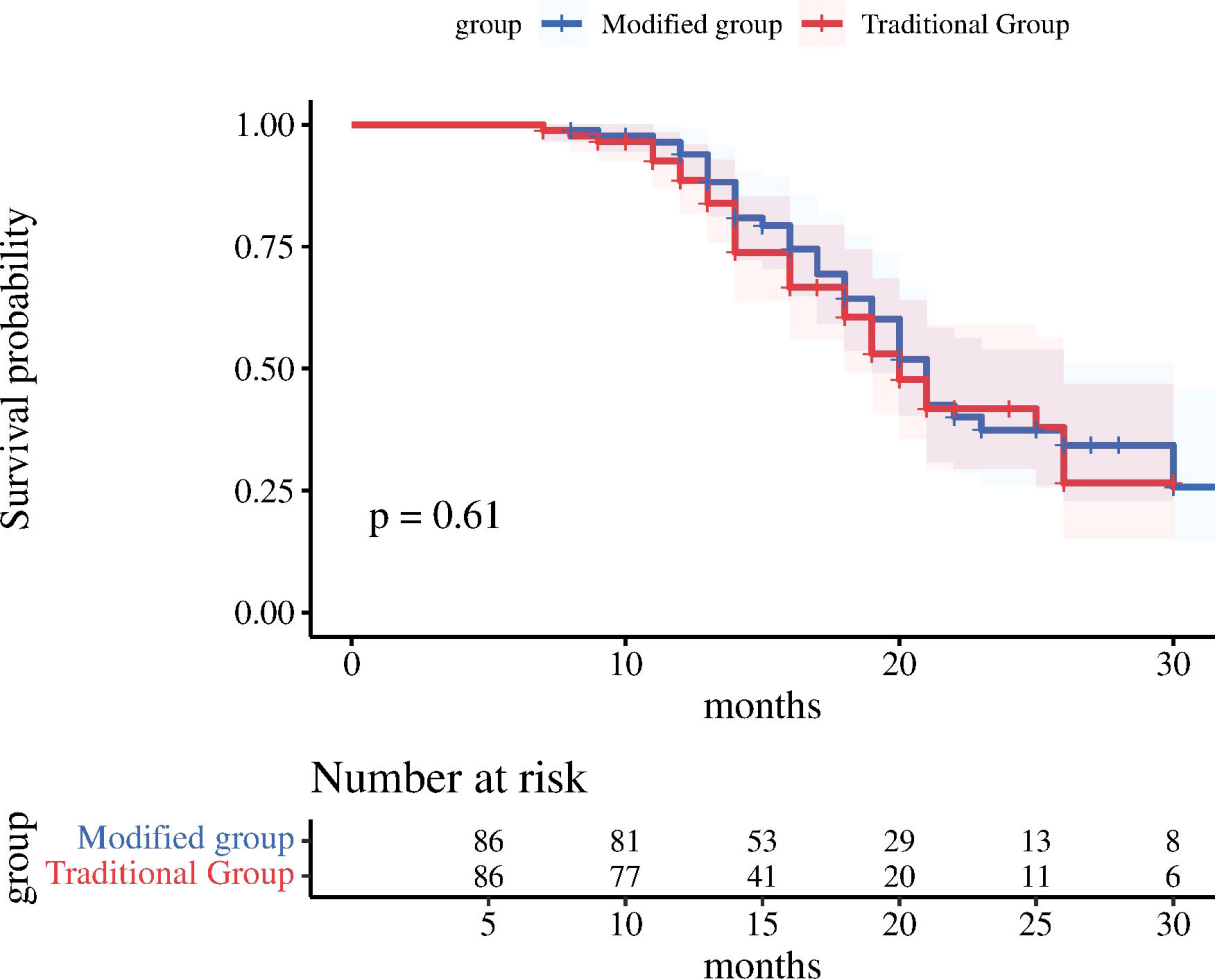
OS survival analysis curves for both groups.

The follow-up was completed in 36 months after surgery for both two groups, and there was no statistically significant difference between the two groups in the comparison of postoperative local recurrence (*p*=0.721) and distant metastasis (*p*=0.742); as displayed in **Table 3**. The median survival time in the modified group was 22.35 months (95% CI: 20.43-24.27); while the median survival time in the traditional group was 21.08 months (95% CI: 19.22-22.94). In the comparison of 3-year OS between the modified and traditional groups (44.2% vs 41.9%; *X* ^2 =^ 0.267, *p*=0.605), the log-rank test for the difference between the two groups was not statistically significant (**Figure 3**).

**Table 3:** Recurrence and metastasis results in both groups.

Observed indicators	Modified group (n=86)	Traditional group (n=86)	*p*
Local recurrence	35 (40.7)	37 (43.0)	0.721
Anastomosis	4 (4.7)	6 (7.0)	
Upper mediastinum	17 (19.8)	12 (14.0)	
Lymph nodes in the neck	10 (11.6)	14 (16.3)	
abdominal cavity	4 (4.7)	5 (5.8)	
Distant transfer	32 (37.2)	33 (38.4)	0.742
Liver	5 (5.8)	7 (8.1)	
Lung	16 (18.6)	10 (11.6)	
Adrenal	6 (7.0)	9 (10.5)	
Bone	4 (4.7)	6 (7.0)	
Multiple metastases	1 (1.2)	1 (1.2)	

## Discussion

With the gradual promotion of minimally invasive surgery and standardized treatment for esophageal cancer, transrectal thoracic surgery for esophageal cancer has been recognized by Chinese thoracic surgeons (9). The thoracoscopic approach through the right chest fully exposes the anatomical structure and tissue adjacent to the esophagus, making it easy to dissect and resect the esophageal tumor, and clear the lymph nodes more thoroughly, with good clinical efficacy (10, 11). However, whole thoracoscopic resection of the azygous vein, thoracic duct and surrounding tissues is difficult and does not easily meet the Ivor-Lewis resection criteria. Therefore, in clinical application, a modified Ivor-Lewis surgical protocol was used to reduce the extent of resection and preserve the azygous vein, thoracic duct, and surrounding tissues; this strategy reduces the difficulty of surgery, while its influence on the long-term outcome is worthy of in-depth investigation.

Our present results showed that the modified group was superior than the traditional group in terms of operative time(*p*=0.007), intraoperative bleeding(*p*=0.003), and postoperative 3 days chest drainage(*p*=0.001) in both two groups, with statistically significant differences. There was no significant difference between the two groups in the aspects of pulmonary infection(*p*=0.732), anastomotic fistula(*p*=0.560), and perioperative mortality, and further, our reported results agree well with previously published results (12). Our results disclosed that both surgical resection or preservation of the azygous vein, thoracic duct and surrounding tissues for esophageal cancer have the same safety, but the preservation of the thoracic duct and azygous vein reduces surgical trauma, saves surgical time, facilitates postoperative recovery, and accords well with the trend of minimally invasive treatment.

Pasquali et al. (13) reported that the metastasis rate of parathoracic duct lymph nodes was 6.6%, and metastasis was related to the depth of tumor infiltration, and the metastasis rate of parathoracic duct lymph nodes was 2.2% for T1b to T2 and 10% for T3 to T4. The preservation of the azygous vein and thoracic duct may not reveal adequately and affect the complete clearance of parathoracic duct lymph nodes. In this study, lymph node metastasis was detected in 2 patients in the traditional group, with a metastasis rate of 2.3%, which is lower than previous value of 6.6%, as reported by Pasquali et al. (13). In these 2 patients, the absence of resection means that the tumor remains and a recent local recurrence of the tumor may occur. Compared with the modified Ivor-Lewis procedure, the traditional Ivor-Lewis procedure has some obvious advantages of thoroughly removing the thoracic duct, the azygous vein, and surrounding tissue, and thus, theoretically reducing the potential for local recurrence in this area. However, the differences in 3-year postoperative survival rates, tumor recurrence types and local recurrence rates among the two groups were not statistically significant based on long-term follow-up results. Therefore, our results showed that few cases of local recurrence are directly related to the surgical modification. Furthermore, Schröder et al. (14) also pointed out that the preservation of the thoracic duct and the azygous vein hasn’t increased local recurrence. It makes clear that the same long-term clinical outcome can be obtained by preserving the thoracic duct, the azygous vein and the surrounding tissue. The effectiveness of preoperative neoadjuvant therapy for esophageal cancer has been proven, and neoadjuvant radiotherapy combined with surgery can prolong patient survival (15, 16). Chinese clinical guidelines also recommend preoperative neoadjuvant radiotherapy for progressive esophageal cancer, and the intervention of preoperative radiotherapy may compensate for the potential risk of residual tumor due to the preservation of the thoracic duct, azygous vein, and surrounding tissue.

Our present study is of some limitations and shortcomings: (i) possible bias in the results due to the single-center data source of the included studies; (ii) despite PSM has been applied to control confounding factors among groups, the potential selection bias has not been eliminated completely. (iii) although the 3-year OS results of the two methods in this study are similar, the long-term oncology effect still needs more research to explore. In addition, a few patients did not undergo positron emission tomography (PET)/CT due to poor economic conditions. Despite these limitations, our study provides insight into the improvement of thoracolaparoscopic Ivor-Lewis esophageal cancer resection.

## Conclusion

The modified way of preserving the thoracic duct and the azygous vein in esophageal surgery reduces the scope of surgery and surgical trauma compared with the classical Ivor-Lewis resection, which is in line with the trend of minimally invasive treatment and does not affect the long-term outcome. Our findings suggest that the thoracic duct, azygous vein and surrounding tissues may not be removed when the tumor does not invade the tissue during the surgery of esophageal cancer. We look forward to large sample randomized controlled trials to verify these results in the future.

## Data availability statement

The raw data supporting the conclusions of this article will be made available by the authors, without undue reservation.

## Ethics statement

The studies involving human participants were reviewed and approved by the Ethics Committee of Gansu Provincial People’s Hospital. The patients/participants provided their written informed consent to participate in this study. Written informed consent was obtained from the individual(s) for the publication of any potentially identifiable images or data included in this article.

## Author contributions

Study design: ZH, YG and XB. Data collection: WG and YS. Data analysis: YL, BC and DJ. Drafting the manuscript: ZH and XW. Project supervision: ZH and YG. All authors contributed to the article and approved the submitted version.
