# Corrigendum: Efficacy of modified thoraco-laparoscopic Ivor-Lewis versus traditional thoraco-laparoscopic Ivor-Lewis for esophageal cancer: propensity score-matched analysis

**DOI:** 10.3389/fonc.2023.1216836

**Published:** 2023-06-14

**Authors:** Ziqiang Hong, Wenxi Gou, Yingjie Lu, Xusheng Wu, Yannan Sheng, Baiqiang Cui, Xiangdou Bai, Dacheng Jin, Yunjiu Gou

**Affiliations:** ^1^ The First Clinical Medical College of Gansu University of Chinese Medicine, Gansu Provincial Hospital, Lanzhou, China; ^2^ Department of Thoracic Surgery, Gansu Provincial Hospital, Lanzhou, China; ^3^ School of Public Health, Southern Medical University, Guangzhou, China

**Keywords:** esophageal cancer, Ivor-Lewis, thoraco-laparoscopy, propensity score matching analysis, modified

In the published article, an author name was incorrectly written as **Ziqaing Hong**. The correct spelling is **Ziqiang Hong**.

The authors apologize for this error and state that this does not change the scientific conclusions of the article in any way. The original article has been updated.

